# Protein Kinase G1 α Overexpression Increases Stem Cell Survival and Cardiac Function after Myocardial Infarction

**DOI:** 10.1371/journal.pone.0060087

**Published:** 2013-03-25

**Authors:** Linlin Wang, Zeeshan Pasha, Shuyun Wang, Ning Li, Yuliang Feng, Gang Lu, Ronald W. Millard, Muhammad Ashraf

**Affiliations:** 1 Laboratory Medicine, Department of Pathology, College of Medicine, University of Cincinnati, Cincinnati, Ohio, United States of America; 2 Department of Pharmacology and Cell Biophysics, College of Medicine, University of Cincinnati, Cincinnati, Ohio, United States of America; Brigham and Women's Hospital, United States of America

## Abstract

**Background:**

We hypothesized that overexpression of cGMP-dependent protein kinase type 1α (PKG1α) could mimic the effect of tadalafil on the survival of bone marrow derived mesenchymal stem cells (MSCs) contributing to regeneration of the ischemic heart.

**Methods and Results:**

MSCs from male rats were transduced with adenoviral vector encoding for PKG1α (^PKG1α^MSCs).Controls included native MSCs (^Nat^MSCs) and MSCs transduced with an empty vector (^Null^MSCs). PKG1α activity was increased approximately 20, 5 and 16 fold respectively in ^PKG1α^MSCs. ^PKG1α^MSCs showed improved survival under oxygen and glucose deprivation (OGD) which was evidenced by lower LDH release, caspase-3/7 activity and number of positive TUNEL cells. Anti-apoptotic proteins pAkt, pGSK3β, and Bcl-2 were significantly increased in ^PKG1α^MSCs compared to ^Nat^MSCs and ^Null^MSCs. Higher release of multiple prosurvival and angiogenic factors such as HGF, bFGF, SDF-1 and Ang-1 was observed in ^PKG1α^MSCs before and after OGD. In a female rat model of acute myocardial infarction, ^PKG1α^MSCs group showed higher survival compared with ^Null^MSCs group at 3 and 7 days after transplantation as determined by TUNEL staining and sry-gene quantitation by real-time PCR. Increased anti-apoptotic proteins and paracrine factors *in vitro* were also identified. Immunostaining for cardiac troponin I combined with GFP showed increased myogenic differentiation of ^PKG1α^MSCs. At 4 weeks after transplantation, compared to DMEM group and ^Null^MSCs group, ^PKG1α^MSCs group showed increased blood vessel density in infarct and peri-infarct areas (62.5±7.7; 68.8±7.3 per microscopic view, p<0.05) and attenuated infarct size (27.2±2.5%, *p*<0.01). Heart function indices including ejection fraction (52.1±2.2%, *p*<0.01) and fractional shortening (24.8%±1.3%, p<0.01) were improved significantly in ^PKG1α^MSCs group.

**Conclusion:**

Overexpression of PKG1α transgene could be a powerful approach to improve MSCs survival and their angiomyogenic potential in the infarcted heart.

## Introduction

Transplantation of bone marrow-derived mesenchymal stem cells (MSCs) has been extensively used to treat cardiovascular ischemic disease due to MSCs' regenerative potential in both experimental animal model and human [1], [2], [3], [4], [5], [6], [7], [8]. Despite of promising results, myriad problems including massive cell death after transplantation have limited the efficacy of this cell therapy [9], [10], [11]. One strategy to improve MSCs survival is to precondition MSCs before transplantation [4], [12], [13]. There are different ways to precondition MSCs including exposing cells to physical treatments (e.g. hypoxia, heat shock), pharmacological agents, “priming” with growth factors, and genetic modification by over-expression of anti-apoptotic proteins, growth factors or pro-survival genes [14], [15], [16], [17].

cGMP-dependent protein kinase (PKG), a critical mediator of cGMP signaling in cardiovascular system, phosphorylates many intracellular proteins to regulate important physiological functions such as cell differentiation and proliferation, and cell survival [18], [19], [20], [21]. PKG has two isozymes, PKG1 and PKG2. Only PKG1 is detected in cardiac myocytes and vascular cells. The N-terminus of PKG1 is encoded by two different exons resulting in the production of two isoforms, PKG1α and PKG1β. Both isoforms are present in heart and vessels [22], [23], [24]. However, PKG1β is activated at ∼10-fold higher cGMP concentrations than PKG1α. Previous studies have revealed a direct role for PKG1 in cardioprotection. Overexpression of PKG1α by adenoviral vectors inhibits necrosis and apoptosis in rat cardiomyocytes undergoing simulated ischemia-reoxygenation[25]. PKG1-deficient mice showed increased myocardial structural and functional damage after trans-aortic constriction (TAC) than their wild-type controls [26]. PKG is also a critical mediator in phosphodiesterase (PDE) inhibitor induced cardioprotection. Treatment with PDE-5 inhibitors, sildenafil or tadalafil, effected cardioprotection both *in vitro* and *in vivo* through PKG1 [27], [28], [29]. In a previous study we showed that PDE5a inhibition with adenoviral short hairpin RNA could protect cardiomyocytes against anoxia, attenuate infarction size and improve cardiac remodeling and dysfunction [30].

Interestingly, the PDE-5 inhibitors can protect MSCs and adipose-derived stem cells (ASCs) in ischemic rat hearts both *in vitro* and *in vivo* via promoting PKG activity [31], [32]. As PKG1α is more active than PKG1β at cGMP each found in the cardiovascular system [25], [33], [34], [35], we hypothesized that overexpression of PKG1α via adenovirus could prolong the survival of MSCs both *in vitro* and *in vivo* in rats with regional myocardial ischemia and improve the cardiac function.

## Materials and Methods

### Ethics Statement

All animal experimental procedures conform to the Guide for the Care and Use of Laboratory Animals published by the US National Institutes of Health (NIH Publication #85-23, revised 1996) and were conducted according to a protocol approved by the Institutional Animal Care and Use Committee, University of Cincinnati.

### In vitro studies

#### Construction of Adenoviral vector of PKG1α

pShuttle-IRES-hrGFP-1 vector containing PKG1α cDNA amplified from mouse cardiomyocytes (Ad-PKG1α) was constructed based on AdEasy™ XL Adenoviral Vector System from Strategene (Agilent Technologies, USA). To prevent restriction enzymes from inappropriately digesting DNA, we introduced site-specific mutation into PKG gene T→C change at position 1773 by using QuickChange Lightning Multi Site-Directed Mutagenesis Kit (Agilent Technologies). This is a non-sense mutation coding for isoleucine. A vector without the therapeutic gene (Ad-null) was routinely made to use as a control. Ad-PKG1α and Ad-null viral vectors were propagated in AD-293 cells using Dulbecco's modified Eagle's medium (DMEM; GIBCO Invitrogen) supplemented with 10% fetal bovine serum. The cell suspension was collected, purified, and used in further experiments.

#### Isolation of MSCs and Adenoviral Transduction

Bone marrow-derived MSCs were isolated from 8–10 week male Fisher rats, as described previously by us [5]. The cells were cultured for no more than 5 passages before using for both *in vitro* and *in vivo* studies. MSCs were transduced with Ad-null or Ad-PKG1α or treated with DMEM for 7 h followed by maintenance in the viral vector-free DMEM for 72 h. Transfection efficiency was about 45%–50%. The successful transduction was judged by the presence of green cells. Randomly selected microscopic fields (n>8; 400×) at 72 h after transduction were evaluated to calculate the ratio of green cells to the total number of cells in several independent experiments. These cells were further confirmed by immunostaining of GFP in either Ad-null transduced MSCs (^Null^MSCs) or Ad-PKG1α transduced MSCs (^PKG1α^MSCs). PKG1α transgene overexpression in ^PKG1α^MSCs was confirmed by reverse transcription polymerase chain reaction (RT-PCR), Western blot and PKG activity assay.

#### Reverse transcription polymerase chain reaction (RT-PCR) and real-time PCR

We used RT-PCR to detect PKG1α gene overexpression and real-time PCR to detect pro-survial and angiogenic cytokines eg. HGF, bFGF, SDF-1, Ang-1 and expression of cardiac transcription factors. Isolation of total RNA from the different groups of MSCs and subsequent first-strand cDNA synthesis was performed using an RNeasy mini kit (Qiagen) and an Omniscript Reverse Transcription kit (Qiagen), respectively following the instructions of manufacturer. The thermocycle profile for PCR was set for initial denaturation at 94°C for 3 min, 30 cycles of denaturation at 94°C for 30 sec, annealing at 58°C for 30 sec and extension at 72°C for 30 sec, and a final extension at 72°C for 10 min. Real-time PCR was performed using iQ SYBR-Green supermix (Bio-Rad) in a Bio-Rad iQ5 optical module. The cycling conditions were set for 3 min at 95°C 15 min for initial denaturation; 40 cycles of denaturation at 94°C for 15 sec, annealing at 55°C for 30 sec, extension at 72°C for 30 sec; and a final extension at 55°C for 15 sec. The data was acquired during the extension step. Melting curves were obtained at the end of the reaction by gradually raising the temperature by 1°C/min from 55°C to 90°C over a time period of 35 minutes. The primer sequences used are shown in Table-1.

**Table 1:** Sequence of the primers used in the study.

Gene Symbol	Primer sequence	Product size(bp)
PKG1α	5′-GCGGAGCCGCAGACCTACAG-3′	224
	5′-ACCAGTGACCCCACATCGCCT-3′	
HGF	5′-CGAGCTATCGCGGTAAAGAC-3′	165
	5′-TGTAGCTTTCACCGTTGCAG-3′	
TGFβ	5′-ACCAACTACTGCTTCAGCTCCACA-3′	166
	5′-TGTACTGTGTGTCCAGGCTCCAAA-3′	
bFGF	5′-AAGAGCGACCCACACGTCAAACTA-3′	110
	5′-AGCCGTCCATCTTCCTTCATAGCA -3′	
SDF-1	5′-CTTTGTGCTGGCAAATCTCA-3′	219
	5′-TGTGCATTGACCCGAAATTA-3′	
Angiopoietin-1	5′-CAGCACAAAGGACGCTGATA-3′	232
	5′-ATAGCGCCTTCAGAAGTCCA-3′	
Nkx2.5	5′-ACCGCCCCTACATTTTATCC-3′	230
	5′-GACAGGTACCGCTGTTGCTT-3′	
GATA4	5′-TCTGGCTGGCCGAGAGCAGT-3′	148
	5′-GGCTGTGCAGGACTGGGCTG-3′	
β-actin	5′-CTCTTCCAGCCTTGCTTCCT-3′	165
	5′-CTTCTGCATCCTGTCAGCAA-3′	

#### PKG activity assay

PKG activity in MSCs was measured by using the Cyclex Cyclic GMP-dependent protein kinase Assay Kit (catalog no. CY-1161; MBL) following the manufacturer instructions.

#### Oxygen glucose deprivation (OGD)

MSCs were seeded at a cell density of 2×10^4^ cells/cm^2^. The cells were divided into three groups, including nontreated native MSCs (^Nat^MSCs), transduced with Ad-null (^Null^MSCs), and transduced with Ad-PKG1α (^PKG1α^MSCs). At 72 h after transduction, MSCs in all groups were rinsed twice with serum-free, glucose, and sodium pyruvate-free DMEM and were cultured in the same medium at 37°C in a anoxia chamber (InVivo 500; Ruskinn Life Science) saturated with 95% N_2_/5% CO_2_ for up to 8 h.

#### LDH release assay

The release of lactate dehydrogenase (LDH) from cells with a damaged membrane after OGD was measured by using Homogeneous Membrane Integrity Assay (Promega) to determine cell viability. The fluorescence readings were obtained with excitation and emission filters at ∼550 and ∼590 nM, respectively. Percentage of cell death was calculated as 100× (Reading_Experimental_-Reading_Background_)/(Reading_Total Release_-Reading_Background_).

#### Caspase-3/7 activity assay

Caspase-3/7 activity was measured by Apo-ONE Homogeneous Caspase-3/7 Assay Kit (Promega) according to manufacturer's instruction. MSCs w ere seeded in a 96-well white-walled plate at a concentration of 10^4^ cells/well in 100 µl medium. After 8 h OGD, a 100 µl of detection buffer was added to a well and the plate was read with a spectrofluorometer at excitation wavelength 485 nm and emission wavelength 535 nm (Plate Chameleon, HIDEX).

#### In vitro apoptosis assay

Terminal dUTP nick-end labeling (TUNEL) assay was performed in three groups of MSCs described above to evaluate OGD induced apoptosis. After exposure of OGD for 8 h, the cells were fixed in 4% paraformaldehyde. TUNEL was performed by using an *in situ* cell death detection kit (TMR Red; Roche Applied Science) according to manufacturer's instructions. The nuclei were visualized by 4′6-diamidino-2-phenylindole (DAPI) in mounting medium. Randomly selected microscopic fields (n = 8) were evaluated to calculate the ratio of TUNEL^+^ cells to the total number of cells in three independent experiments.

#### Western blot analysis

Protein lysate samples from cultured cells were obtained with ice cold lysis buffer (50 mM Tris-HCl, PH 7.6, 120 Mm NaCl, 0.5% NP-40, 1 mM PMSF, 2 µg/ml Leupeptin, and 2 µg/ml Aprotinin). Protein concentration was measured by using the Bio-Rad DC-Protein Assay Reagent (Bio-Rad). 20 µg of protein samples were loaded onto 4%-12% gradient precast gel (Invitrogen) and electrophoresed for 2 h at 100 Volts. Transblotting was performed onto a Polyvinylidene Fluoride (PVDF) membrane as described before [36]. The membrane was blocked with 5% nonfat milk in Tris-buffered saline with Tween (TBST) for 1 h and subsequently incubated with specific primary antibodies (diluted in 5% nonfat milk or bovine serum albumin (BSA) in TBST) for overnight at 4°C. After washing with TBST 3 times, the membrane was incubated with horseradish peroxidase (HRP)-conjugated secondary antibodies for 1 h at room temperature. After washing with TBST, membranes were incubated with ECL or Femto supersensitive detection reagent, and their signals were exposed to Kodak Light film (Fisher Scientific). The primary antibodies used in Western blot included anti PKG1α (Santa Cruz, USA, 1∶200), anti-phosphorylated Akt (pAkt) (Cell Signaling Technology, USA 1∶2000), anti Akt (Cell Signaling Technology, USA 1∶2000), anti-phosphorylated GSK3β (pGSK3β) (Cell Signaling Technology, USA 1∶2000), anti GSK3β (Cell Signaling Technology, USA 1∶2000), anti Bcl2 (Cell Signaling Technology, USA 1∶2000).

#### Cell proliferation assay

We used Ki67 immunostaining to evaluate cell proliferation in MSCs. After 8 h OGD, the cells were fixed in 4% paraformaldehyde. Immunostaining was routinely performed. The cells were then mounted on slides which were exposed to the medium containing (DAPI). Randomly selected microscopic fields (n = 8) were evaluated to calculate the ratio of Ki67 positive cells to the total number of cells in three independent experiments.

### In vivo studies

#### Experimental rat model of acute myocardial infarction and cell transplantation

MSCs from GFP transgenic male rats were used to study the fate of transplanted cells. An experimental rat model of acute myocardial infarction in young female Fischer rats (180–200 g) was developed by permanent ligation of the coronary artery, as described before [5].The number of stem cells injected varied in different studies. Massive donor cell death after transplantation is one of the key problems which need to be resolved in stem cell therapy. The number of cells to be given to the animal depends on the route of injection in order to maximize the protective effect. As previously reported from other research groups, 5-6×10^6^ [37], [38] were successfully injected in the rat hearts to determine their effects. We have previously injected 3×10^6^ MSCs [31]at multiple sites in and around the infarct area to maximize the efficacy of cell therapy. In the current study, we determined that 2×10^6^ was the most suitable number for transplantation without any side effects.

Rats (n = 16/group) received intramyocardial injections of either 70 µl of DMEM without cells (DMEM group), or containing 2×10^6^ ^Null^MSCs (^Null^MSCs group), or containing 2×10^6^ ^PKG1α^MSCs (^PKG1α^MSCs group). The injections were given at multiple sites (4–5 sites/animal) in and around the infarcted area. The animals were euthanized at stipulated time points (n = 4 per group on day 3 and n = 8 per group at 4 weeks) for histological and molecular studies.

#### Molecular Analyses

For molecular analysis, the animals were euthanized at day-3 after stem cell transplantation. Left ventricle was cut and processed for PKG1α expression and activity assay, paracrine and cardiac transcription factors expression and caspase-3/7 activity assay. pAkt, Akt, pGSK3β, GSK3β and Bcl-2 were measured by Western blot. To determine the survival of transplanted male MSCs, sry-gene analysis was performed at day-7 after stem cell transplantation by using isolated gDNA from left ventricle tissues.

#### Immunohistological studies

TUNEL staining was performed on heart tissue sections for transplanted MSCs (combined with GFP) and residual cardiomyocytes (combined with desmin) at day 3 after stem cell transplantation. Double immunostaining of cardiac troponin I (TnI; 1∶100, Santa Cruz, USA) and GFP (1∶800, Abcam, USA) was performed at 7 days after stem cell transplantation.

At 4 weeks after transplantation, infarction size was measured in paraffin embedded heart tissue sections with Mason's trichome staining by using computer-based planimetry with Image-J analysis software (version 1.6065; NIH).

Blood vessels were identified by immunostaining of vonWillebrand Factor-VIII (vWF-VIII, 1∶ 200; Dako, Denmark) and detected with fluorescently labeled secondary antibody (Molecular Probes). The number of blood vessels were counted in both infarct and peri-infarct regions. At least 48 microscopic fields each in infarct and peri-infarct regions (n = 8) were randomly selected and counted in each group. Blood vessel density was expressed as the number of vessels per microscopic view at 200× magnification.

#### Heart function measurement

At 4 weeks after stem cell transplantation, transthoracic echocardiography was used to measure the heart function as described before [5], [39]. Indices of left ventricle (LV) systolic function including LV fractional shortening (LVFS) and LV ejection fraction (LVEF) were calculated respectively. The results were expressed as percentage. LV chamber dimensions (end-diastolic dimension [EDD] and end-systolic dimension [ESD]), and anterior wall thickness were measured.

#### Statistical analysis

All data were expressed as mean ± SE. Student's t-test or one-way ANOVA was performed to analyze statistical differences in each response variable. A value of *p*<0.05 was defined as statistically significant.

## Results

### In vitro studies

#### Transgenic overexpression of PKG1α in MSCs

**Figure 1:**
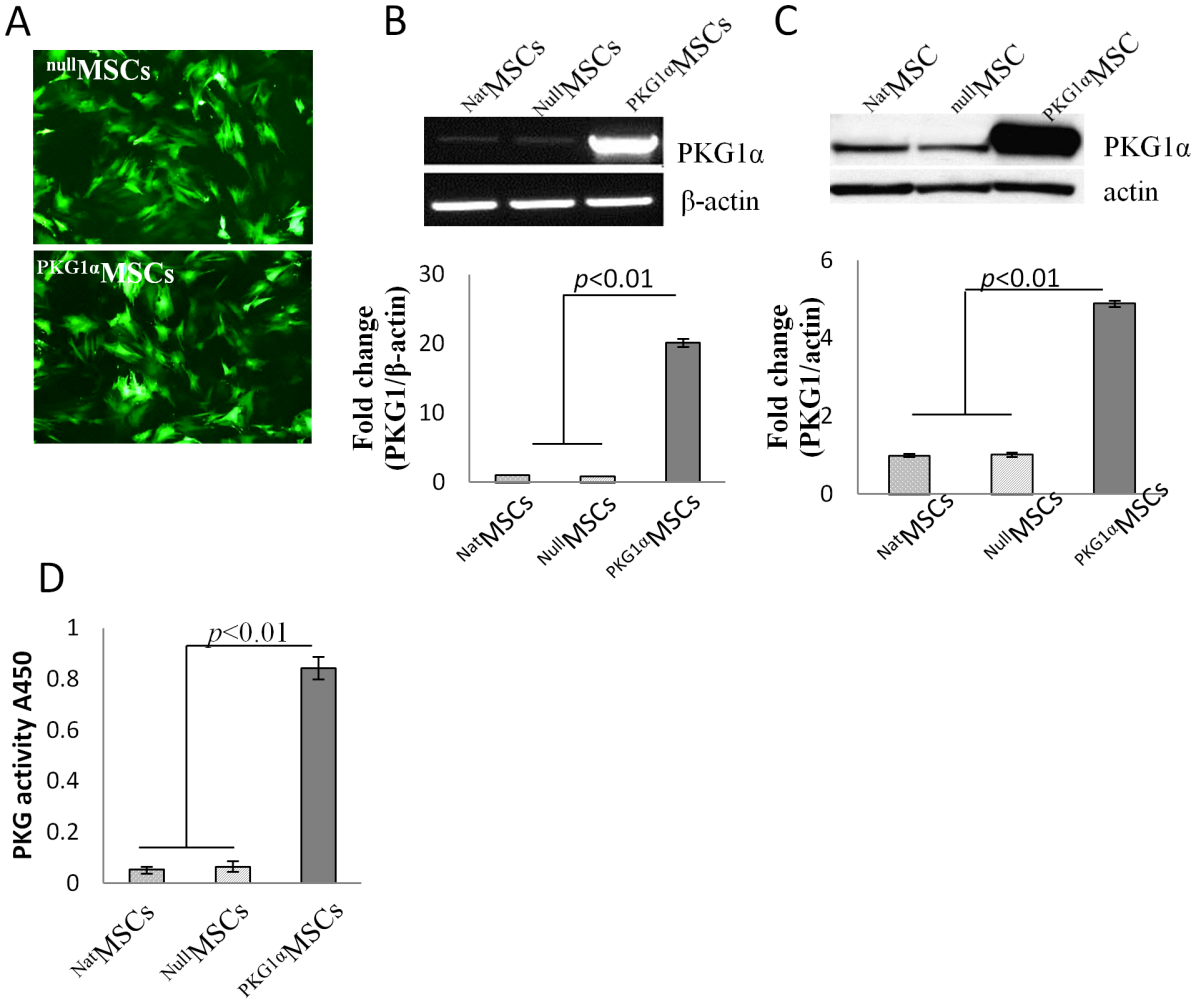
PKG1α overexpression in MSCs. (A) Fluorescence microscopic images (green) of MSCs successfully transducted with Ad-null and Ad-PKG1á 48 h after transduction; (B) RT-PCR showed enhanced mRNA level of PKG1á in ^PKG1á^MSCs; (C) western blot showed higher PKG1á and PKG1á-flag fusion proteins in ^PKG1á^MSCs. (D) PKG activity increased 16 fold in ^PKG1á^MSCs compared to ^Nat^MSCs.

Adenovirus transduction was evidenced by GFP (green fluorescence) expression in ^Null^MSCs and ^PKG1α^MSCs (Fig. 1A). Compared to ^Nat^MSCs and ^Null^MSCs, ^PKG1α^MSCs had significantly higher PKG1α gene, protein expression and PKG activity (Fig.1B–D, p<0.01).

#### PKG1α promoted MSCs survival and proliferation under OGD

**Figure 2:**
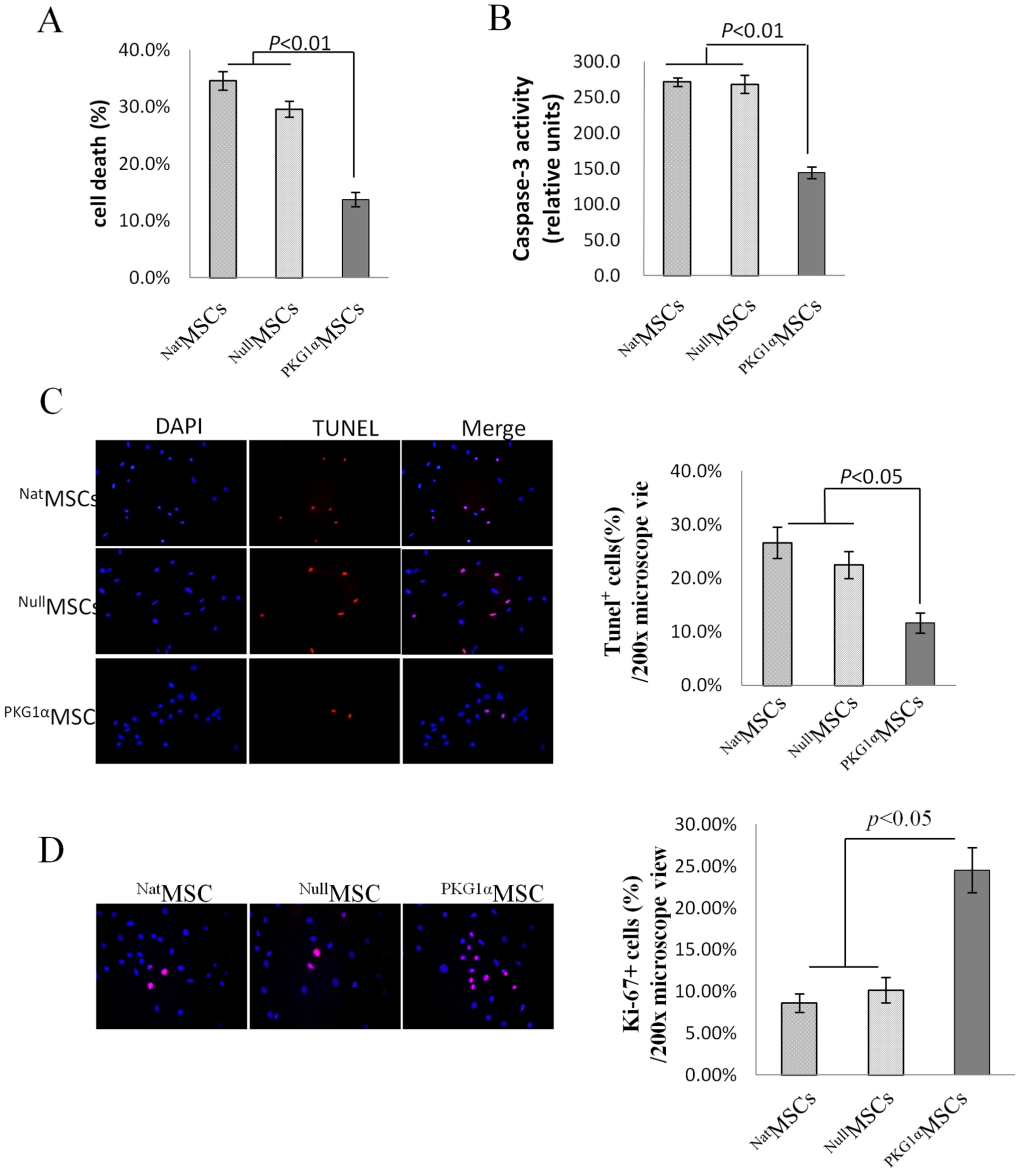
Effect of PKG1α overexpression on MSCs survival. (A) LDH release assay showed less cell death on ^PKG1α^ MSCs at 8 h after exposure to oxygen glucose deprivation (OGD); (B) Parallel experiments were performed to show that caspase-3/7 activity was significantly decreased in ^PKG1α^MSCs with concomitant increase in cell survival as determined by LDH release assay; (C–D) TUNEL and Ki67 staining on MSCs showed decreased number of TUNEL(+) cells and increased Ki67(+) cells in ^PKG1α^ MSCs compare with ^Nat^MSCs and ^Null^MSCs after 8 h OGD. The nuclei were visualized by staining with DAPI (blue). Magnification ×400.

Compared to controls (^Nat^MSCs and ^Null^MSCs), ^PKG1α^MSCs had a reduced cell damage after 8 h OGD shown by low level of LDH release (^PKG1α^MSCs 13.7±1.3% *vs* ^Nat^MSCs 34.6±1.6% and ^Null^MSCs 29.6±1.4%, *p*<0.01) (Fig. 2A), decreased caspase-3/7 activity (*p*<0.01)(Fig. 2B) and reduced number of TUNEL positive cells (11.7±1.9% *vs* 22.5±2.5% and 26.6±2.9%, *p*<0.05) (Fig. 2C). These results clearly showed the cytoprotective effects of PKG1α transgene on MSCs under glucose and serum-free anoxic conditions. In terms of cell proliferation, there were more Ki67 positive cells seen in ^PKG1α^MSCs (41.2%±3% and 24.5±2.6%) than in controls (^Nat^MSCs 17.8%±2% and 8.6±1.1%; ^Null^MSCs 18.6%±3% and 10.2±1.5%) both before and after OGD (*p*<0.05) (Fig. 2D).

#### PKG1α overexpression reduced apoptosis

**Figure 3:**
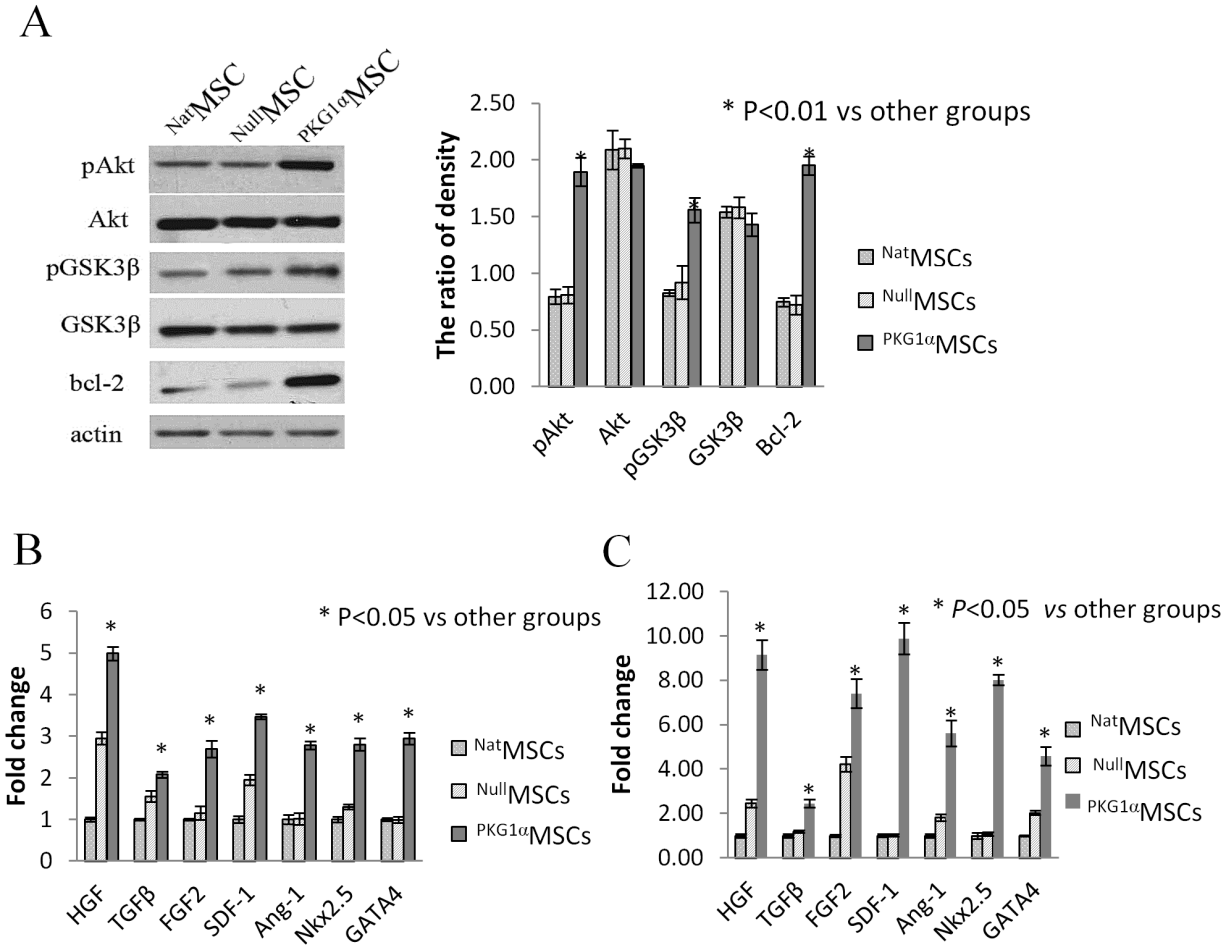
PKG1α overexpression activated pro-survival pathway in vitro. (A) Increased phosphorylation of Akt,GSK3â and high level of Bcl-2 expression were detected in ^PKG1á^ MSCs by western blot after 48 h OGD, protein expression of pAkt, pGSK3â and Bcl-2 are 2.4, 1.7, 2.8 fold higher respectively as compared to ^Nat^MSCs without changing the total level of Akt and GSK3â (*p*<0.01). (B and C) Real-time PCR based gene array analysis showed several pro-survival and angiogenic factors including HGF, TGFâ, FGF2, SDF-1 and Ang-1 as well as 2 cardiac transcription factors Nkx2.5 and GATA4 genes significantly increased as compared with the control cells (n = 3 experiments) before or after OGD (*p*<0.05).

In order to delineate the pro-survival pathway in ^PKG1α^MSCs, we isolated and analyzed the cell lysate from different cell treatment groups for Western blot. PKG1α overexpression significantly increased phosphorylation of Akt (pAkt) and GSK3β (pGSK3β) without changing the total level of Akt and GSK3β in ^PKG1α^MSCs after 8 h OGD compared to controls (^Nat^MSCs and ^Null^MSCs) (Fig. 3A). GSK3β is a downstream target in Akt signaling. Anti-apoptotic protein Bcl-2, was also significantly increased in ^PKG1α^MSCs (Fig. 3A).

#### PKG1α increased multiple pro-survival and angiomyogenic factors

Compared to controls (^Nat^MSCs and ^Null^MSCs), ^PKG1α^MSCs had significant pro-survival, angiogenic and cardiac transcription factors before and after OGD. Higher expression of paracrine factors including HGF, bFGF, SDF-1 and Ang-1 (p<0.01) and cardiac transcription factors Nkx2.5, GATA-4 (p<0.01) were documented before and after OGD (Fig. 3B). Moreover, ^PKG1α^MSCs showed more than 2-fold increase in gene expression of these factors after OGD (Fig. 3C).

### In vivo studies

#### PKG1α expression in ^PKG1α^MSCs transplanted hearts

**Figure 4:**
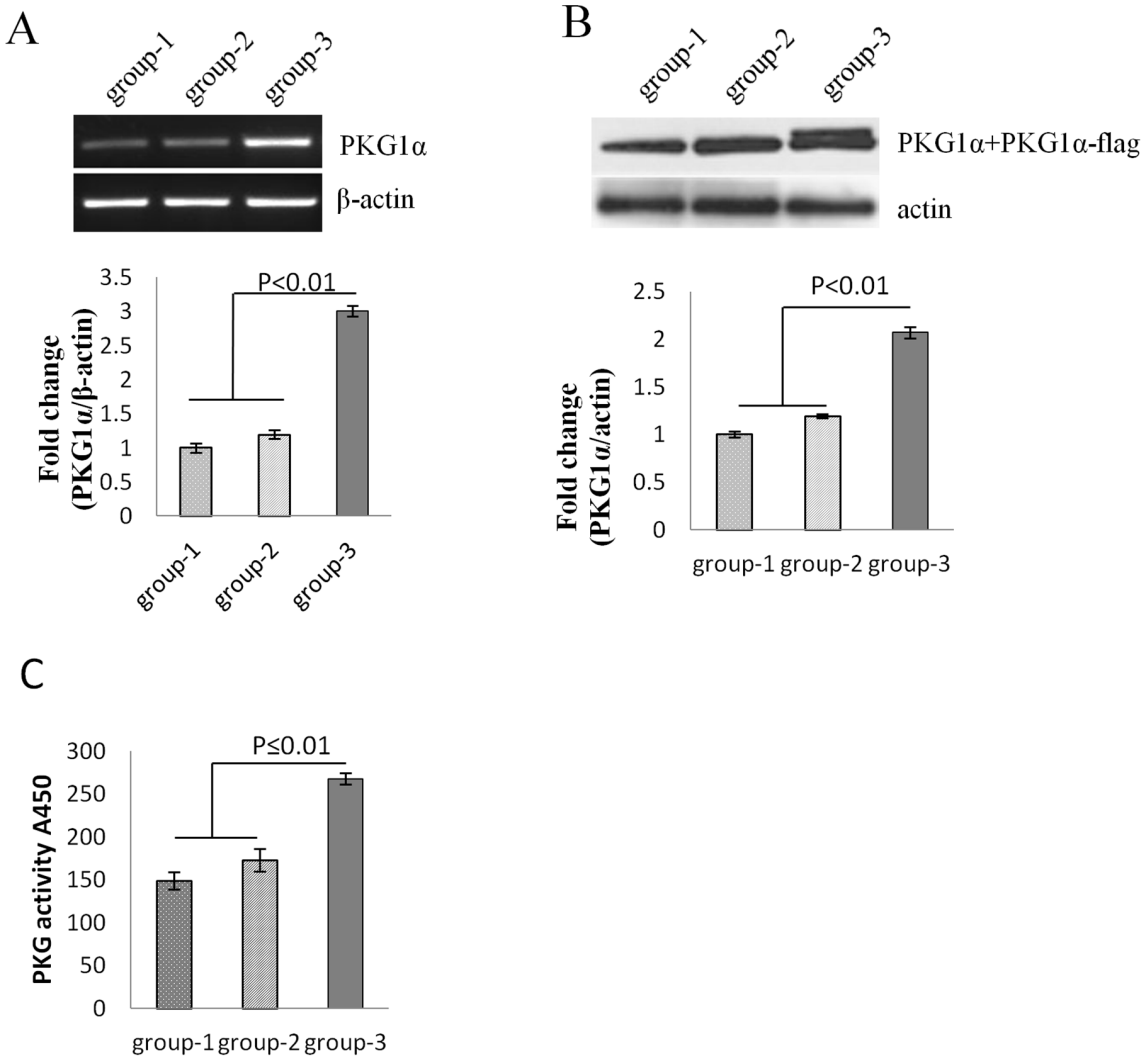
PKG1α overexpression in vivo. (A) RT-PCR showed a significant increase in PKG1á mRNA level in group-3 (*p*<0.01); (B) PKG1á and transducted PKG1á-flag fusion proteins were detected by western blot and showed 2 fold higher expression in group-3 as compared to group-1 and group-2 (*p*<0.01). (C) PKG activity was enhanced 1.6 fold after ^PKG1á^MSCs transplantation (*p*<0.01). Rat hearts injected with DMEM (group-1); injected with ^Null^MSCs(group-2); injected with ^PKG1á^MSCs (group-3).

All animals after their respective treatment in different groups survived the full length of the experiment (4 weeks), and there were no animal deaths related to PKG1α transgene overexpression in the infarcted heart. Analysis of the LV tissue samples by RT-PCR showed that the transplanted cells continued to overexpress PKG1α at the site of the cell graft in rat hearts. The level of PKG1α transgene expression on day 3 was elevated in ^PKG1α^MSCs group compared to DMEM and ^Null^MSCs group (Fig. 4A). PKG1α protein expression (Fig. 4B, *p*<0.01) and PKG activity were also significantly increased in ^PKG1α^MSCs group (Fig. 4C).

#### PKG1α improved survival of transplanted MSCs and cardiomyocytes in rats with acute myocardial infarction

**Figure 5:**
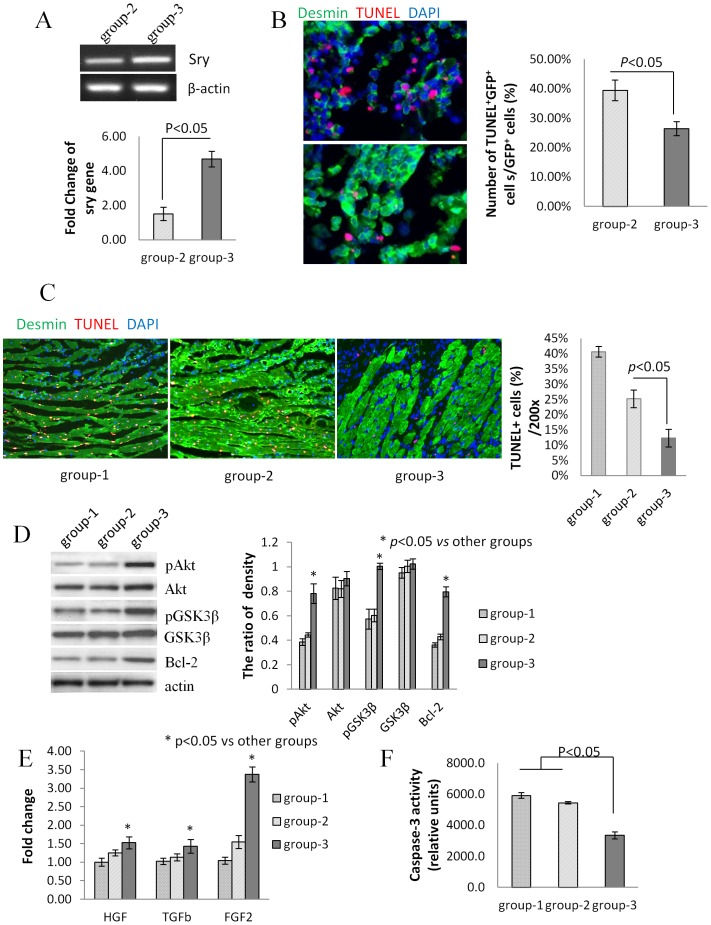
Cardioprotective effects of transduced PKG1α in vivo. (A) Real-time PCR for sry gene in female rat heart model of acute myocardial infarction at 7 days after sex-mismatched transplantation of ^Null^MSCs (group-2) and ^PKG1α^MSCs (group-3). Cell survival was significantly higher in ^PKG1α^MSCs as compared with the ^Null^MSCs group (n = 4/group). (B) TUNEL and GFP double staining on histological sections of rat heart tissue injected with ^Null^MSCs (group-2) and ^PKG1α^MSCs (group-3). The number of apoptotic transplanted MSCs (TUNEL^+^GFP^+^, red and green) was significantly reduced in group-3 (*p*<0.05 *vs* group-2). (C) TUNEL(red) combined with desmin immunostaining (green) on histological sections showed less apoptosis in residual myocardium in group-3 compared to group-1 and group-2 (*p*<0.05). (D) Western blot on protein lysate samples of the rat left ventricle showed increased phosphorylation of Akt,GSK3β, and high level of Bcl-2 expression at 3 days after transplantation in group-3 (p<0.05, n = 4/group). (E) Real-time PCR for growth factors expression showed HGF, TGFβ and FGF2 were significantly increased in left ventricle tissue at 3 days after transplantation. (F) Diminished caspase-3 activity was detected in heart tissues in group-3.

DMEM group did not show any amplification of sry-gene as there were no transplanted male MSCs. Compared to ^Null^MSCs group, ^PKG1α^MSCs group had a significantly higher survival rate (Fig. 5A) based on the sry-gene analysis, and had fewer TUNEL^+^ cells amongst transplanted MSCs (Fig. 5B) and cardiomyocytes in or around infarct regions (Fig. 5C). Higher caspase-3/7 activity was detected in DMEM and ^Null^MSCs group compared to ^PKG1α^MSCs group (Fig. 5E). Expression of anti-apoptosis proteins including pAkt, pGSK3β and Bcl-2 (Fig. 5D), and pro-survival and angiogenic factors including HGF, bFGF, SDF-1 and Ang-1 (p<0.01) (Fig. 5F) were significantly increased.

#### ^PKG1α^MSCs promoted myoangiogenensis in the infarcted rat hearts

**Figure 6:**
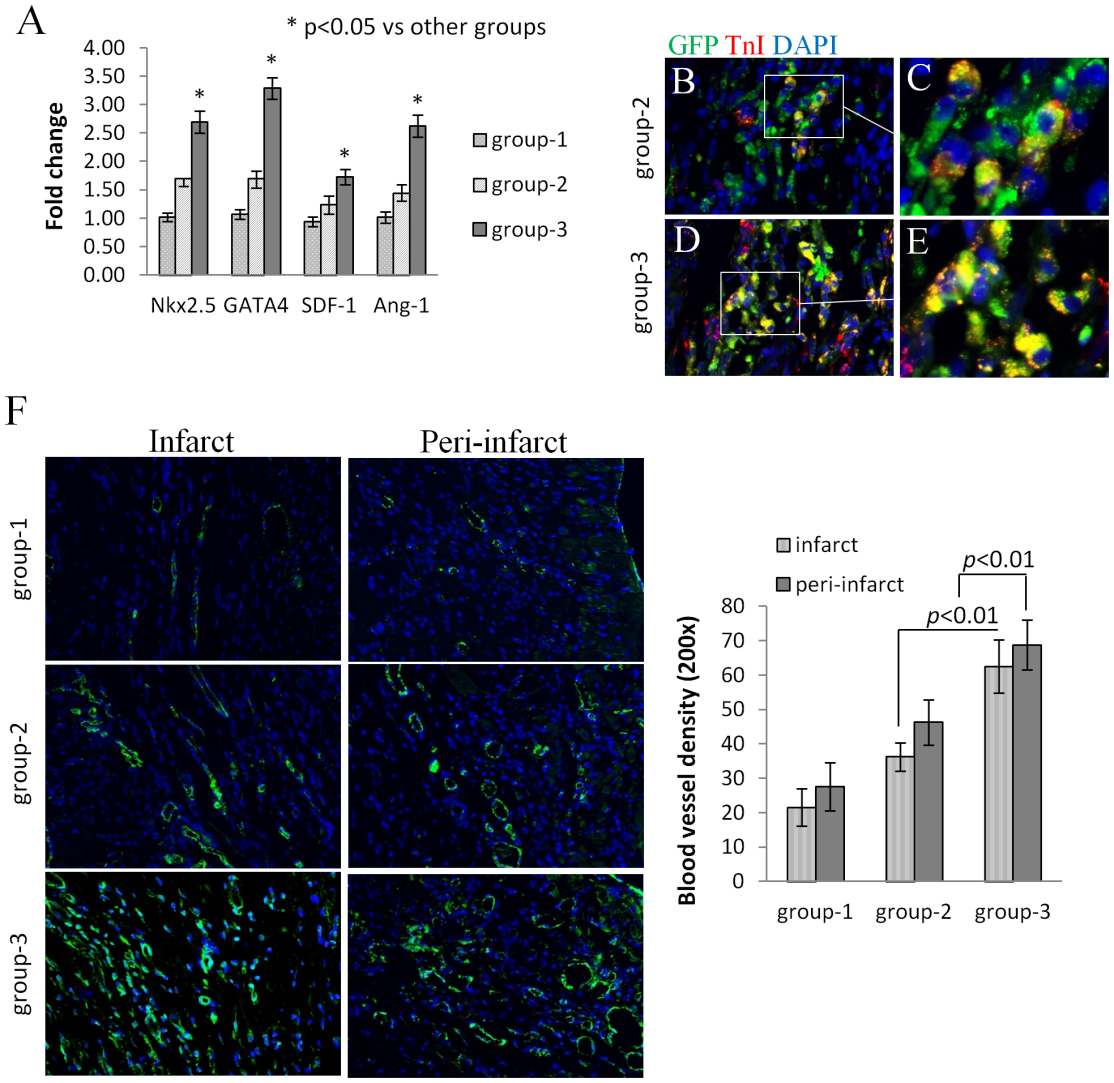
^PKG1α^MSCs promoted angiomyogenesis in the infarct hearts. (A) Real time PCR showed a significant increase in cardiac transcription factor Nkx2.5, GATA4 and pro-angiogenic factor SDF-1, Ang-1 genes expression in left ventricle tissue at 3 days after transplantation. (B–E) Double immunostaining for troponin I (red) and green fluorescent protein (GFP, green) on histological sections showed transplanted ^PKG1α^MSCs differentiated into neofibers more actively as compared to ^null^MSCs at 7 days after transplantation. The nuclei were visualized by staining with DAPI. (E) Fluorescence immunostaining of histological sections from group-2 and group-3 for von Willebrand Factor VIII (green) at 4 weeks after transplantation, blood vessel density was significantly higher in both infarct and peri-infarct regions in group-3 as compared with group-1 and group-2(P<0.01).

At day 3 after transplantation, upregulation of cardiac transcription factors NKx2.5 and GATA-4 were observed in left ventricle tissue of rats treated with ^PKG1α^MSCs compared to DMEM and ^Null^MSCs (*p*<0.01) (Fig. 6A). Double-fluorescence immunostaining for cardiac troponin I (TnI, red) and GFP (green) at day 7 after transplantation showed more neomyofibers in transplanted hearts with ^PKG1α^MSCs than in ^null^MSCs treated hearts (Fig. 6B–D).

Rat hearts transplanted with ^PKG1α^MSCs also showed extensive angiogenic response as compared to DMEM and ^Null^MSCs group. Blood vessel density was significantly higher in the infarct and peri-infarct areas in ^PKG1α^MSCs group (62.5±7.7 and 68.8±7.3 per microscopic view, p<0.05) compared to DMEM group (21.5±5.4 and 27.5±7.0 per microscopic view) and ^Null^MSCs group (36.3±4.1 and 46.3±6.6 per microscopic view) after 4 weeks of transplantation (Fig. 6F).

#### ^PKG1α^MSCs improved cardiac function and attenuated infarction size

**Figure 7:**
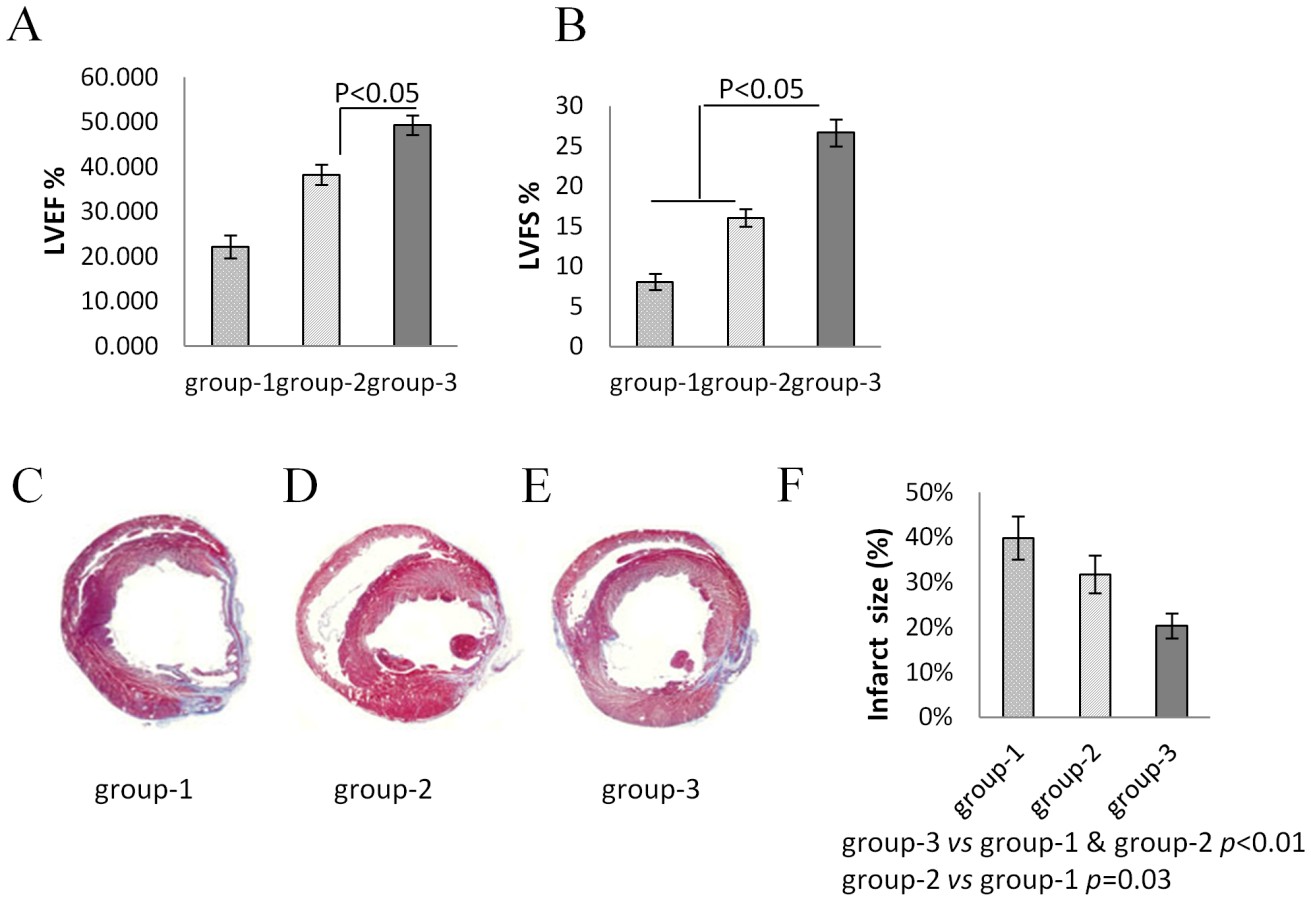
^PKG1α^MSCs improved cardiac function and attenuated infarction size. At 4 weeks after transplantation, (A and B) transthoracic echocardiography was performed to assess global heart function. Both LV ejection fraction (LVEF) and fractional shortening (LVFS) were significantly preserved in group-3 compared to group-1 and group-2 (*p*<0.05). (C–F) Quantitative assessment on the rat heart sections with mason trichrome staining showed a significant attenuation of infarct size in group-3 compared with group-1 and group-2 (*p*<0.01) (n = 8/group).

At 4 weeks after transplantation, transthoracic echocardiography showed that the indices of LV heart function, including left ventricle ejection fraction (LVEF) and left ventricle fractional shortening (LVFS), were significantly preserved in ^PKG1α^MSCs group (LVEF 52.1±2.2%; LVFS 24.8±1.3%) compared to DMEM group (LVEF 22.4±1.2%, *p*<0.01; LVFS 8.1±0.5%, *p*<0.01) and ^Null^MSCs group (LVEF 38.4±2.4%, *p*<0.05; LVFS 15.2±1.5% *p*<0.01) (Fig. 7A–B). The baseline values of LVEF and LVFS were 91.4±1.3% and 60.5±1.5%. Both LV end-diastolic dimension (LVEDD) and end-systolic dimension (LVESD) (in millimeters) were smaller in ^PKG1α^MSCs group (LVEDD 7.5±0.3, LVESD 5.8±0.3; *p*<0.01) compared to DMEM group (LVEDD 8.9±0.5, LVESD 8.1±0.3) and ^Null^MSCs group (LVEDD 8.0±0.4, LVESD 6.8±0.4). Histological sections showed the infarction size was significantly reduced in ^PKG1α^MSCs group (20.2±2.5%, *p*<0.01) compared to DMEM (44.8±4.3%) and ^Null^MSCs group (30.1±3.7%). Improved LV wall thickness was also observed in ^PKG1α^MSCs group (p<0.05 *vs* DMEM and ^Null^MSCs group) (Fig.7C–F).

## Discussion

The major finding of the present study is that the overexpression of PKG1α transgene in MSCs significantly enhanced their resistance to ischemic stress and angiomyogenic potential in the infarcted heart. To our knowledge, this is the first time that the direct overexpression of the PKG1α transgene has been shown to effectively promote the survival of transplanted MSCs' via an anti-apoptotic mechanism and a coordinated increase of angiogenesis by paracrine factors in ischemic hearts.

One critical issue of stem cell therapy is how to reduce massive donor cell death after transplantation. *In vitro* preconditioning strategies have been used to enhance survival of transplanted MSCs in the infarcted heart. PKG1 was considered as a key effector in cardioprotection induced by PDE-5 inhibitors against ischemic injury in the infarcted heart and cardiomyocytes [27], [28], [29], [31], [32]. In a previous study we demonstrated that the PDE-5 inhibitor tadalafil could promote MSCs survival in the infarcted heart via activating PKG1 [31]. However, little is known about the direct effect of PKG1α on protecting MSCs and improving cardiac function. Our present study revealed that overexpression of PKG1α directly by adenoviral transduction could prolong the survival of MSCs against ischemic stress both *in vitro* and in infarcted hearts *in vivo*, and preserve cardiomyocyte survival and cardiac function following ischemic injury. Because of the prolonged survival of MSCs under ischemic stress both *in vitro* and *in vivo*, the cardiac function was preserved. These findings are consistent with previous results confirming tadalafil induced protection by PKG1α and suggests a new approach to improve stem cell therapy following MI in patients.

The potential mechanism of overexpression of PKG1α could include its anti-apoptotic effect as is evident by increased phosphorylation of Akt (pAkt) and GSK3β (pGSK3β), Bcl-2 expression, and prevention of caspase-3/7 activation. This is consistent with a previous report from Das et al, that showed higher upregulation of pAkt, pGSK3β and Bcl-2 played an essential role in the protective effect of PKG1α on cardiomyocytes in the infarcted heart [25], [27].

Although stem cell transplantation can protect ischemic cardiac muscle, reduce infarct size, and preserve cardiac function, the mechanisms are still unclear. Our present study demonstrated that overexpression of PKG1α in MSCs was responsible for the release of multiple paracrine factors. The significant increases in HGF, bFGF and SDF-1 detected at an early stage (day 3) of transplantation revealed their important role in angiogenesis, anti-apoptosis and sparing of damaged cardiomyocytes in cardiac repair [40], [41], [42], [43]. The secreted paracrine factors were prominent within 72 h after transplantation and affected the cellular milieu for stem cell retention and survival. They provide the early benefit to the infarcted heart which may eventually trigger other pathways leading to protection and regeneration by endogenous stem cells [44]. Our results are supported by the previous study where sildenafil preconditioned adipose-derived stem cells (ASCs) expressed higher growth factors including bFGF, HGF and Ang-1 and were associated with significant reduction in infarct size, cardiomyocytes apoptosis and improve heart function following ischemia [32]. Taken together, our study provided multiple potential pathways that could explain the link between prolonged stem cell life, cardiac protection and functional recovery. Direct regulation of these cytokines could become another target of gene therapy for survival of stem cells.

Preconditioning is also believed to enhance the differentiation potential of stem cells. More neomyocytes (TnI and GFP double positive cells) were observed in ^PKG1α^MSCs transplanted hearts than those transplanted with ^Null^MSCs. In addition, elevated expression of cardiac transcription factors Nkx2.5 and GATA-4 was detected both in vitro and in vivo by real-time PCR. Although transplantation of ^PKG1α^ MSCs led to a significant angiomyogenic response, it could be the result of regeneration by both exogenous stem cells and endogenous stem cells activated by paracrine factors released by ^PKG1α^MSCs in agreement with a previous report [44].This suggests that PKG1α gene delivery improved the myogenic potential of MSCs. The difference between ^Null^MSCs and ^PKG1α^ MSCs in their myogenic differentiation post transplantation in the heart may also be due to the inhibition of GSK3β. Previous studies have shown that inhibition of GSK3β is sufficient to stimulate myogenic differentiation [45], [46]. We observed increased phosphorylation of GSK3β as well as Akt in ^PKG1α^ MSCs after OGD and in ^PKG1α^MSCs transplanted hearts following infarction compared to ^Null^MSCs. From these observations we propose that PKG1α gene delivery promotes the myogenic potential of MSCs by phosphorylating and inactivating GSK3β activity via PI3K/Akt pathway and contributing to the cardiac recovery after ischemia.

Despite the positive data, our study has several limitations. First, we only studied the biomarker and paracrine factors in MSCs transplanted hearts till 7 days after transplantation. Further observation is necessary to determine the long term cell fate of PKG1α preconditioned MSCs and overexpression of cytokines, growth factors, anti-apoptotic and angiogenic factors as well as the formation of neomyocytes and blood vessles in the infarcted hearts. Second, an extensive and in-depth analysis of the secretable growth factors other than angiogenic and anti-apoptosis factors, which are up-regulated in ^PKG1α^ MSCs may be of value. Third, although transplantation of ^PKG1α^ MSCs led to a significant angiomyogenic response, we did not determine the functional status of the newly formed blood vessels and myofibers. Lastly, our previous study provided the evidence that PDE-5 inhibition with tadalafil prolonged survival of transplanted MSCs in ischemic heart via cGMP/PKG signaling. Future studies are needed to compare the effects of preconditioning with PDE5 inhibitors *vs* direct PKG1α gene delivering in the same experimental settings for cytoprotective and cardioprotective effects.

### Conclusions

In conclusion, direct PKG1α transgene overexpression via adenoviral transduction significantly improves MSCs survival under conditions of ischemia via anti-apoptotic mechanism and promotes their angiomyogenic potential in ischemic heart. The preconditioned MSCs attenuated ischemia induced cardiomyocyte apoptosis, reduced the heart infarct size and improved the global cardiac function. Our findings suggest that direct PKG1α gene expression could be a new therapeutic target in stem cell-based therapy for the ischemic heart disease.
